# Influence of Carbamazepine Dihydrate on the Preparation of Amorphous Solid Dispersions by Hot Melt Extrusion

**DOI:** 10.3390/pharmaceutics12040379

**Published:** 2020-04-20

**Authors:** Xiangyu Ma, Felix Müller, Siyuan Huang, Michael Lowinger, Xu Liu, Rebecca Schooler, Robert O. Williams

**Affiliations:** 1 Molecular Pharmaceutics and Drug Delivery, College of Pharmacy, The University of Texas at Austin, 2409 University Avenue, Austin, TX 78712, USA; roderickma@outlook.com (X.M.); felix.mue@hotmail.de (F.M.); xliu@utexas.edu (X.L.); becca@theschoolers.com (R.S.); 2 Small Molecule Design and Development, Lilly Research Laboratories, Eli Lilly and Company, Indianapolis, IN 46221, USA; huang_siyuan@lilly.com; 3 Merck Research Laboratories, Merck & Co., Inc., 126 E. Lincoln Ave, Rahway, NJ 07065, USA; mike.lowinger@gmail.com

**Keywords:** Amorphous solid dispersions, hot melt extrusion, hydrate, anhydrate, dehydration

## Abstract

Amorphous solid dispersions (ASDs) are commonly used in the pharmaceutical industry to improve the dissolution and bioavailability of poorly water-soluble drugs. Hot melt extrusion (HME) has been employed to prepare ASD based products. However, due to the narrow processing window of HME, ASDs are normally obtained with high processing temperatures and mechanical stress. Interestingly, one-third of pharmaceutical compounds reportedly exist in hydrate forms. In this study, we selected carbamazepine (CBZ) dihydrate to investigate its solid-state changes during the dehydration process and the impact of the dehydration on the preparation of CBZ ASDs using a Leistritz micro-18 extruder. Various characterization techniques were used to study the dehydration kinetics of CBZ dihydrate under different conditions. We designed the extrusion runs and demonstrated that: 1) the dehydration of CBZ dihydrate resulted in a disordered state of the drug molecule; 2) the resulted higher energy state CBZ facilitated the drug solubilization and mixing with the polymer matrix during the HME process, which significantly decreased the required extrusion temperature from 140 to 60 °C for CBZ ASDs manufacturing compared to directly processing anhydrous crystalline CBZ. This work illustrated that the proper utilization of drug hydrates can significantly improve the processability of HME for preparing ASDs.

## 1. Introduction

The increasing number of poorly water-soluble drug candidates continues to present challenges to pharmaceutical product development [1]. Reportedly, 90% of drugs in the development phase and 40% of marketed products exhibit poor water solubility [2,3,4,5]. Researchers have demonstrated that amorphous solid dispersions (ASD) can enable the delivery of poorly water-soluble drugs for fast dissolution and improved bioavailability [6]. In the last two decades, ASDs have gained popularity in the pharmaceutical industry, and their importance to solving drug solubility challenges for enhancing bioavailability of drugs is exemplified by the fact that 27 ASD based products have been approved by the Food and Drug Administration (FDA) [5].

Various techniques such as spray drying [7], hot melt extrusion (HME) [8], Kinetisol^®^ dispersion [9], and thin film freezing [10] have been developed and investigated to prepare ASDs. As one of the commercially used techniques, HME has been applied in the pharmaceutical industry to 14 ASD based products [5]. HME process provides mechanical energy (shear and pressure) [11] and thermal energy (heat transfer) [12] to enable the melting of the drug and polymer, particle size reduction, mixing between components, and forming of the extrudates [13,14]. The energy provided during the HME process is necessary to achieve complete amorphization of drugs and homogenous mixing between the drug and excipients [12]. Due to the rheological properties of polymers used in HME, processing temperatures and screw speeds need to be maintained at a relatively high level in order to enable the extrusion process. However, the excessive amount of energy generated by the extruder can lead to the chemical degradation of drug molecules and excipients, which is detrimental to the product development [13,15]. Therefore, the energy generated during the HME process must be carefully controlled to minimize any chemical degradation in the resulting ASDs [13]. Also, there is a need to expand this narrow processing window of HME to allow its applications for more poorly water-soluble drugs in the pharmaceutical industry [16].

More than one-third of pharmaceutical compounds can form hydrates [17], and in some cases, drug hydrates are more stable and preferred compared to the corresponding anhydrous form [18,19]. Those hydrates exhibit various physicochemical properties depending on the extent of interactions between water molecules and drug crystalline lattice [20]. For example, water molecules may be integrated into the crystalline structure or weakly bonded to the drug molecules [20]. It is well known that those hydrates can undergo dehydration during processing (e.g., HME [21], granulation [22], milling [23]), which are performed under an elevated temperature, low water vapor pressure, or high level of mechanical stress [24]. The dehydration of those hydrates can result in an unstable amorphous phase [25,26], different anhydrate polymorphs, or an anhydrate form with the identical crystalline lattice as the original hydrate [20,27,28]. Different dehydration pathways of drug hydrates may significantly affect pharmaceutical processes and product quality. For instance, Raijada et al. observed a lower dehydration temperature of nitrofurantoin monohydrate during the HME process where the degree of phase transition was affected by various processing parameters such as temperature, screw speed, and residence time [21].

Interestingly, besides conventional approaches (e.g., HME, spray drying, freeze drying), researchers have demonstrated that the dehydration of drug hydrates can be applied to prepare amorphous drugs for solubility improvement purpose [24,29]. Li et al. successfully prepared amorphous carbamazepine (CBZ) through the in-situ dehydration of CBZ dihydrate [28]. Further studies illustrate that by precisely controlling the environmental conditions (e.g., water vapor pressure less than 7.6 Torr), CBZ dihydrate dehydrates into a metastable amorphous phase, which can be achieved at a lower temperature (40 °C) compared to the melting temperature of CBZ anhydrate (192 °C, form-I) [30,31]. We hypothesize that this decreased amorphization temperature may have been beneficial to the CBZ ASD preparations that Huang et al. reported on regarding the chemical degradation of CBZ anhydrate during HME process [32]. However, the resulting amorphous phase of CBZ from dehydration is not thermodynamically stable and may crystallize into a stable anhydrous polymorph at a higher temperature and water vapor pressure [33,34]. For example, Horstman et al. investigated the feasibility of using twin-screw extruders to prepare amorphous drugs at a large scale [35]. However, due to the instability of the amorphous phase following the dehydration, a mixture of crystalline anhydrates in different polymorphs was obtained after the extrusion process [35]. Alternatively, a conventional HME process with a fast quench of the molten drug (melting temperature at 200 °C) was used to achieve the production of amorphous drugs with minimal chemical degradation [35].

In this study, CBZ dihydrate was selected as the model drug, and Soluplus^®^-Vitamin E succinate was used as the polymer matrix. A Leistritz Micro-18 extruder was used to process CBZ dihydrate and polymers. We hypothesized that the utilization of CBZ dihydrate during the extrusion process could significantly improve the processability of CBZ ASDs. We investigated the effects of CBZ dihydrate on the preparation of CBZ ASDs by HME and demonstrated that the utilization of in-situ dehydration of CBZ dihydrate can significantly reduce the temperature required for amorphization during the HME process, which expands the processing window of HME and provides useful insights of drug form selection.

## 2. Materials and Methods

### 2.1. Materials

Carbamazepine (CBZ) of form-III was purchased from Letco Medical (Decatur, AL, USA) [30,36]. Soluplus^®^ was donated by the BASF Corporation (Florham Park, NJ, USA). Vitamin E succinate was purchased from VWR International, LLC., (Radnor, PA, USA). Acetonitrile, methanol, and water (HPLC grade) were purchased from Fisher Scientific Co. (Houston, TX, USA). Other chemicals used in this study were of American Chemical Society (ACS) grade. Chemical structures of form-III CBZ anhydrate, Soluplus^®^, and Vitamin E succinates are shown in Supporting Information Figure S1.

### 2.2. Preparation of Carbamazepine (CBZ) Dihydrate

CBZ dihydrate was prepared by suspending form-III CBZ anhydrate in distilled water and stirring for 48 h. The slurry was filtered using suction filtration, and the resulting solids were dried by lyophilization at 0 °C and 2500 mTorr for 6 h. The formation of CBZ dihydrate was confirmed by various characterization techniques described in the following sections. Dried CBZ dihydrate was stored at room temperature and 56% relative humidity. Carbamazepine exists in five anhydrous polymorphs (form-I T_melting_ at 192 °C, form-II T_phase transition_ at 135–170 °C, form-III T_melting_ at 175 °C, form-IV T_melting_ at 187 °C, and form-V) and a dihydrate form [37,38,39]. Generally, the order of stability of those four polymorphs under ambient conditions is: form-III CBZ anhydrate > form-I > form-IV > form-II [37]. CBZ dihydrate can be prepared using all those four anhydrous forms [40,41]. The conversion kinetics of those polymorphs to CBZ dihydrate were dependent on the crystal morphology of different polymorphs [40]. Researchers have demonstrated that the morphology of CBZ dihydrate obtained from form-III CBZ anhydrate or form-I CBZ anhydrate was comparable [41]. Therefore, in this study, form-III CBZ anhydrate was used to prepare CBZ dihydrate since the received neat CBZ was in form-III CBZ anhydrate. Interestingly, CBZ dihydrate was reportedly prepared using two different methods, the water-suspension approach and the water/ethanol crystallization method [42,43]. In our study, both methods were tested to prepare CBZ dihydrate. However, different DSC thermograms of the prepared CBZ dihydrate for those two methods were observed (Supporting Information Figure S2). The CBZ dihydrate obtained from the water-suspension method was dehydrated at a lower temperature of 40–80 °C. Meanwhile, the water/methanol approached resulted in a higher dehydration temperature around 65–100 °C without the observation of recrystallization. Briefly, the dehydration of CBZ dihydrate led to a state of disordered CBZ molecules, which subsequently recrystallized into a mixture of form-III CBZ anhydrate to form-I CBZ anhydrate. However, for the water/ethanol method, no phase transition was observed, and only a melting peak of form-I CBZ anhydrate was shown at 192 °C. Interestingly, X-ray powder diffraction (XRPD) patterns of these the CBZ dihydrate prepared by both methods were comparable (results were not shown), which was also confirmed by others [43]. The possible explanations for the discrepancy found between the dehydration of CBZ dihydrate from these methods were discussed in other literature. Briefly, the water-suspension method resulted in agglomerated forms of which the dehydration process was governed by the boundary layer-internal diffusion-controlled mechanism, whereas the water/ethanol CBZ dihydrate was controlled by an internal diffusion mechanism [42,43]. Therefore, we chose the water-suspension method to prepare CBZ dihydrate in this study because of its lower dehydration temperature and an observable disordered state of CBZ molecules following the dehydration of dihydrate.

### 2.3. Karl Fischer Titration (KFT)

The water contents of the prepared CBZ dihydrate and Soluplus^®^-Vitamin E succinate matrices were measured using a C20S Compact Karl Fischer (KF) Coulometer (Mettler-Toledo LCC, Columbus, OH, USA). Samples of 25–30 mg were accurately weighed and fully dissolved in 1 mL anhydrous methanol. The solution was vortexed for 2 min and transferred to a KF vessel. The weight of the solvate added in the KF vessel was calculated according to Sahakijpijarn et al. [44]. The water content of a reference with anhydrous methanol was also measured and subtracted from the sample water content.

### 2.4. Thermal Analysis

#### 2.4.1. Differential Scanning Calorimetry (DSC)

Conventional DSC and modulated DSC (mDSC) were performed to characterize form-III CBZ anhydrate, CBZ dihydrate, physical mixtures of drug and polymers, and extrudates. A TA Instruments Q20 DSC equipped with a RCS40 refrigerated cooling system (TA Instruments, New Castle, DE, USA) was utilized to analyze all samples under a dry nitrogen purge (200 mL/min). An indium standard was used to the enthalpy and temperature calibration. Tzero^®^ (TA Instruments, New Castle, DE, USA) pans were used for reference and samples. Five to six milligrams of samples were weighed accurately and crimped with a pinhole hermetic aluminum lid. Conventional DSC was conducted in triplicate, and samples were held at 20 °C for 5 min followed by a heating process of 10 °C/min up to 200 °C. mDSC was conducted in heat–cool–heat cycles on physical mixtures to determine the miscibility between form-III CBZ anhydrate and polymer matrices. mDSC was set to perform the first heating cycle of 10 °C/min up to 200 °C followed by a cooling step to 20 °C. The samples were then reheated at a rate of 3 °C/min with a temperature modulation of 1 °C every 60 s up to 200 °C.

#### 2.4.2. Thermal Gravimetric Analysis (TGA)

CBZ dihydrate was subject to TGA measurement using a Mettler Thermogravimetric Analyzer (Mettler–Toledo LCC, Columbus, OH, USA). Each sample of 8 to 10 mg was loaded into a standard aluminum pan of 70 μL. Weight loss measurements were conducted at a ramp rate of 10 °C/min up to 120 °C under a dry nitrogen purge of 50 mL/min.

### 2.5. X-ray Powder Diffraction (XRPD)

XRPD was performed to determine the formation of CBZ dihydrate and polymorphisms of CBZ in different samples. A Rigaku Miniflex 600 (Rigaku, Tokyo, Japan) X-Ray Diffractometer equipped with a Cu Kα radiator of 40 kV and 15 mA was used. Diffraction patterns of all samples were collected at a step size of 0.02° and a scanning speed of 1 degree per second over the two-theta range of 5–35°. Analyses of data were performed using Jade 9 software (KS Analytical Systems, Aubrey, TX, USA).

### 2.6. Polarized Light Microscopy (PLM) with Hot Stage

An Olympus BX-53 polarized light microscope (Olympus Corporation of the Americas, Center Valley, PA, USA) equipped with a QImaging QICAM digital camera (QImaging, Surrey, British Columbia, Canada) was used to observe form-III CBZ anhydrate and CBZ dihydrate crystals in different formulations. A Linkam T95 hot-stage system (Linkam Scientific Instrument, Tadworth, UK) was connected to PLM to study the dehydration of CBZ dihydrate at ambient conditions. Samples were heated at a rate of 10 °C/min up to 220 °C. Images were captured every 6 seconds with a resolution of 1392 × 1100 using Linksys 32 software (Linkam Scientific Instrument, Tadworth, UK).

### 2.7. Processing by Hot Melt Extrusion (HME)

Polymer blends containing 70% *w*/*w* Soluplus^®^ and 30% Vitamin E succinate were prepared using a Turbula^®^ mixer (Glen Mills Inc., Clifton, NJ, USA). A co-rotating Leistritz Micro-18 twin-screw extruder (American Leistritz Extruder Corp., Somerville, NJ, USA) was used to prepare the ASDs. A barrel configuration of feeding drugs and polymers separately was applied. Twin-screw volumetric feeders (Brabender Technology, Duisburg, Germany) were used to feed the polymer blends and drugs with a total feed rate of 6 g/min. Two drug loadings, 10% and 25% *w*/*w* CBZ, were achieved by adjusting the feed rate of the drug and polymer blends accordingly. To be noted, the drug loading throughout the study indicates the amount of dried CBZ without hydrated water molecules. Different screw designs were tested to investigate the effects of screw design on the preparation of CBZ ASDs. Formulations using CBZ dihydrate or form-III CBZ anhydrate were prepared at various barrel temperatures (ranging from 60–140 °C) and at two screw speeds (100 and 200 rpm). Extrudates were naturally cooled under ambient conditions. All extrudates were milled at 4000 rpm using a Fitzpatrick L1A Fitzmill equipped with an impact configuration and a 0.033-inch screen (Fitzpatrick, Inc., Elmhurst, IN, USA). Milled powders were collected and stored in a desiccator with calcium sulfate and cobalt chloride indicators for further studies.

### 2.8. High-Performance Liquid Chromatography (HPLC)

The potency of CBZ in the extrudates was analyzed using a Waters HPLC system (Waters Corporation, Milford, MA) equipped with a photodiode array (PDA)-UV detector (Waters 2998 PD detector, Milford, MA, USA). A reverse phase C18 column with specifications of 5 × 20 mm and 5 μm particle size (Thermo Scientific, Waltham, MA, USA) was used and maintained room temperature. Samples were injected using an autosampler at a volume of 10 μL. The UV absorbance of all samples was measured at a wavelength of 263 nm. The HPLC was operated with an isocratic flow rate of 1 mL/min for a 60:40 acetonitrile/water mobile phase. Empower 3 software (Waters Corporation, Milford, MA, USA) was used to analyze the chromatography data.

### 2.9. Intrinsic Dissolution Rates (IDR) Measurement

IDRs of CBZ dihydrate and form-III CBZ anhydrate in methanol-water solutions of different water activities were measured using the rotating disc method described in United States Pharmacopeia (USP). Briefly, 150 mg of form-III CBZ anhydrate or CBZ dihydrate were compressed into a disk of a diameter of 5.7 mm using a hydraulic press (BVA Hydraulics MTCM-1, US, Kansas City) at a pressure of 1000 psi for 1 min. XRPD was used to confirm no changes in the polymorphs of form-III CBZ anhydrate or CBZ dihydrate. The obtained drug disk was examined visually to ensure a smooth surface that was in the same plane as the surface of the die. Various methanol/water dissolution media of 300 mL with different water activities from 0.55 to 0.7 were prepared [45]. Methanol/water dissolution media of 300 mL with 0.5% and 3% *w*/*w* Soluplus^®^ were also prepared at different water activities to study the effects of Soluplus^®^ on the phase boundary between CBZ dihydrate and form-III CBZ anhydrate. The IDR measurements for CBZ dihydrate and form-III CBZ anhydrate were carried out in triplicate using an overhead mixer (Fisher Scientific, Pittsburgh, PA) with a rotation speed of 50 RPM [46] at 25 °C for 10 minutes [45]. The UV absorbance of the solution was measured at 285 nm by a charge-coupled device (CCD)-Array UV-Spectrophotometer Probe (S.I. Photonics, Arizona, Tucson) with a frequency of 2 acquisitions per minute. The absorbance-time data was converted to a mass-time profile based on an external standard curve, which has a concentration of CBZ ranging from 0.5 µg/mL to 30 µg/L.

### 2.10. Rheology Studies

Soluplus^®^/Vitamin E succinate (7:3) physical blends were processed using a HAAKE™ MiniLab twin-screw co-rotating extruder (Thermo Fisher Scientific Inc., Waltham, MA, USA) at 140 °C and 200 RPM screw speed. The resulting extrudates were milled using an IKA^®^ Tube Mill (IKA Works, Inc., Wilmington, NC, USA) at a rate of 20,000 RPM for 1 min. Then, powders were collected for rheological studies. The processed Soluplus^®^/Vitamin E succinate powders were also mixed with 10% *w*/*w* CBZ dihydrate or 10% *w*/*w* form-III CBZ anhydrate using mortar and pestles. A rheometer AR 2000 (TA Instruments, New Castle, DE, USA) was used to characterize rheological properties of the processed Soluplus^®^-Vitamin E succinate samples and physical blends of drug and polymers. Each sample of 1 g was pressed into a disk of 25 mm in diameter and 1 mm in thickness, and subsequently loaded and pre-heated at 50 °C between two parallel plates (25 mm in diameter) for the tests. Oscillation mode was used to measure the complex viscosity of all samples in triplicate for a temperature sweep or angular frequency sweep. Dynamic temperature tests were carried out at a constant angular frequency of 1 rad/s and a ramp rate of 5 °C/min up to 120 °C. Dynamic angular frequency tests were conducted at a constant temperature of 60 °C and a ramp rate from 1 rad/s to 100 rad/s. A strain of 0.05% was applied throughout the experiments to ensure the complex viscosity stay within the linear viscoelastic region of the materials.

### 2.11. Wide Angle X-ray Scattering (WAXS) with Temperature Control

In order to study the dehydration of CBZ dihydrate with the presence of processed polymer matrices, WAXS measurements of CBZ dihydrate, form-III CBZ anhydrate, and physical mixtures of the processed Soluplus^®^-Vitamin E succinate with CBZ dihydrate/form-III anhydrate at different ratios were conducted at 25 °C and 60 °C on a custom-built SAXSLab instrument (SAXSLab, Northampton, MA, USA) at the University of Texas at Austin (Austin, TX, USA). The instrument is equipped with a microfocus Cu k-alpha rotating anode X-ray source operated at 50 kV and 0.6 mA and a PILATUS3 R 300K (DECTRIS Ltd., Philadelphia, PA, USA) detector. The detector is equipped with three detecting modules of 83.8 × 106.6 mm^2^ sensitive area. The pixel size is 172 × 172 μm^2^. The distance between the sample and detector ranged from 0.95 to 1.45 m. Disposable glass capillaries (Hampton Research, Aliso Viejo, CA, USA) of a 0.9 mm outside diameter with a wall thickness of 0.01 mm were used to load samples. Ganesha instrument control center software (SAXSLab, Northampton, MA, USA) was used to control the instrument. The configuration of 2 apertures WAXS and 2 mm off-centered beam stop was used for all measurements. The acquisition time for each sample was set at 300 s with a beam stop mask and correction for the sample thickness of 0.9 mm. All data were corrected for cosmetic background radiation and an incident beam strength by measuring the X-ray intensity directly on the detector. Data analyses were performed using SAXSGUI software (SAXSLab, Northampton, MA, USA).

### 2.12. Study Design and Excipients Selection

In this study, due to the disordered state of CBZ molecules following the dehydration of CBZ dihydrate, we hypothesized that utilization of this phenomenon can facilitate CBZ ASD preparation under moderate extrusion conditions. Additionally, Soluplus^®^ was selected due to its relatively low T_g_ (72 °C) and viscosity of 100,000 pa*s at lower temperatures compared to other polymers used for HME. The preliminary study (results were not shown) also investigated the miscibility of another low T_g_ polymer (Eudragit^®^ EPO, 45 °C) with CBZ. Compared to Eudragit^®^ EPO, Soluplus^®^ exhibited better miscibility with CBZ, which was also confirmed by other researchers [32]. Though Soluplus^®^ exhibited a relatively low glass transition temperature, the complex viscosity of the polymer melts at 60 °C was above 100,000 pa*s, which was much higher than the extrudable range (1000–10,000 pa*s). Therefore, a plasticizer, Vitamin E succinate (T_melting_ at 76 °C), was added to the formulations to enable the extrusion processes at a lower temperature staring from 60 °C. Other commonly used plasticizers such as polyethylene glycol (PEG 8000) or D-α-tocopheryl polyethylene glycol succinate (Vitamin E TPGS) were also tested (results were not shown). Due to the strong interactions between those plasticizers with CBZ, they were not suitable in our study to separately investigate the effects of CBZ dihydrate on the preparation of CBZ ASDs.

## 3. Results

### 3.1. Formation of CBZ Dihydrate

**Figure 1:**
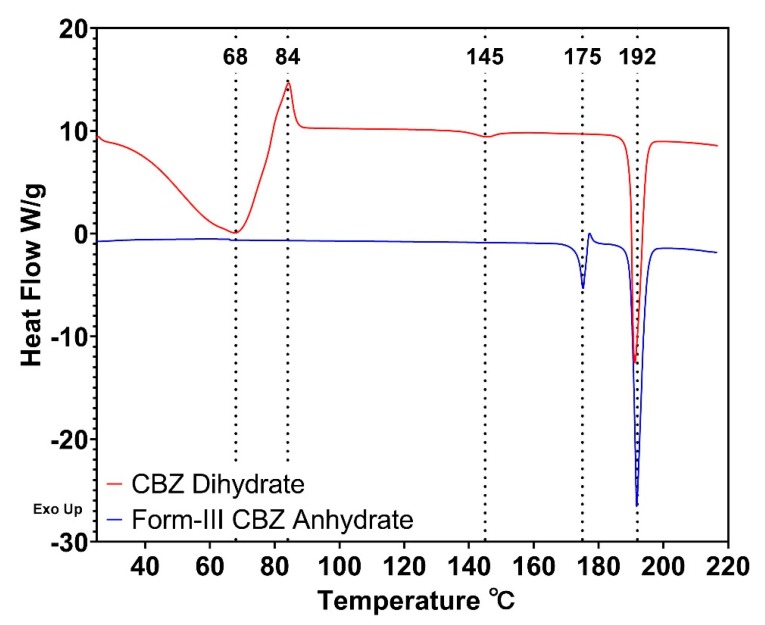
DSC thermograms of CBZ dihydrate (red) and form-III CBZ anhydrate (blue).

The received form-III CBZ anhydrate and prepared CBZ dihydrate were characterized using various techniques. For DSC analysis (Figure 1), form-III CBZ anhydrate exhibited two endothermic peaks and one exothermic peak at 175 °C, 192 °C, and 178 °C, respectively. The endothermic peak at 175 °C corresponds to the melting of form-III CBZ anhydrate followed by the exothermic recrystallization event of CBZ molecules at 178 °C [37]. The recrystallized CBZ was in the form-I, which exhibited a melting peak at 192 °C [37]. CBZ dihydrate prepared using the water-suspension method described in Section 2.2 showed several thermal events. The dehydration of CBZ dihydrate occurred at a temperature ranging from 40 °C to 80 °C. An exothermic peak was observed at 84 °C, which corresponded to the recrystallization of disordered CBZ molecules resulted from dehydration [28]. The endothermic peak at 145 °C was related to the phase transition from form-III CBZ anhydrate to form-I CBZ anhydrate, which melted at 192 °C [41].

**Figure 2:**
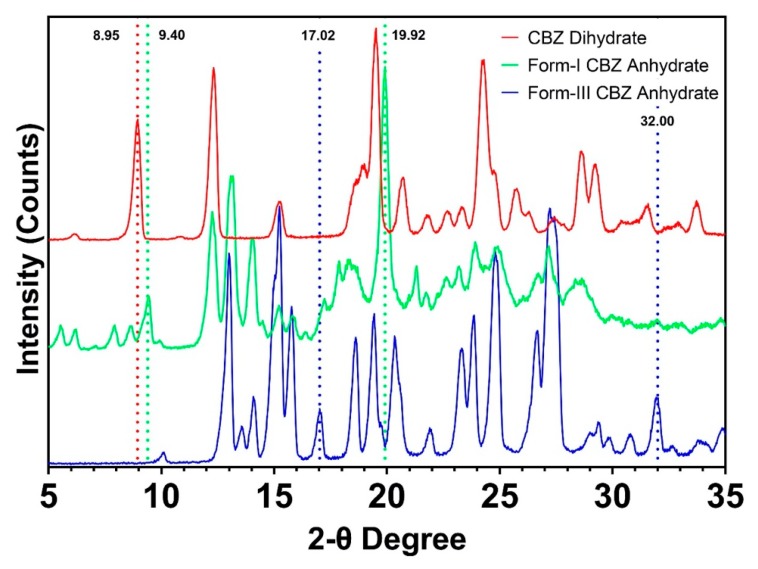
XRPD patterns of CBZ dihydrate (red), form-I CBZ anhydrate (green), and form-III CBZ anhydrate (blue).

CBZ dihydrate, form-III CBZ anhydrate, and form-I CBZ anhydrate were characterized by XRPD, as shown in Figure 2. Form-I CBZ anhydrate was obtained by heating form-III CBZ dihydrate at 150 °C for 3 h [37]. All of three CBZ crystals exhibited distinctive XRPD patterns. Specifically, CBZ dihydrate showed a characteristic peak at 8.95 two-theta degrees. Form-I CBZ anhydrate exhibited unique peaks at 9.40/19.92 two-theta degree, and the peaks at 17.02/32.00 two-theta degrees of form-III CBZ anhydrate were used for polymorphism identification.

Two methods, KF titration and TGA, were used to confirm the formation of CBZ dihydrate (Supporting information, Table S1). KF titration showed a water content of tested CBZ dihydrate of 13.3%, whereas 13.2% weight loss of CBZ dihydrate was observed in TGA. The results corresponded to the theoretical water content of CBZ dihydrate, which is 13.2%. In addition, the processed Soluplus^®^-Vitamin E succinate was heated to 120 °C using TGA, and less than 1% weight loss was recorded. This indicates that the processed Soluplus^®^-Vitamin E succinate has limited moisture content.

Results from those experiments demonstrate the successful preparation of CBZ dihydrate using the water-suspension method. More importantly, the drying approach of using lyophilization for CBZ dihydrate maintained its intact hydrate form without the loss of water molecules from the crystalline lattice.

### 3.2. Dehydration Kinetics of CBZ Dihydrate

**Figure 3:**
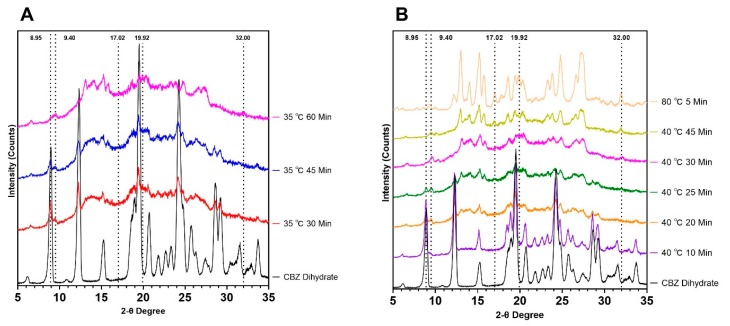
XRPD patterns of dehydrated CBZ dihydrate at 35 °C (**A**) and 40 °C/80 °C (**B**). Three characteristic peaks at 8.95, 17.02/32.00, and 9.40/19.92 two-theta degrees correspond to CBZ dihydrate, form-III CBZ anhydrate, and form-I CBZ anhydrate, respectively.

Dehydration kinetics of CBZ dihydrate was studied using XRPD to provide information for selecting extrusion processing parameters. Neat CBZ dihydrate was heated at 10 °C/min from the room temperature and hold at different temperatures for different periods in the DSC instrument under a dry nitrogen purge with accurate temperature and time control. The heated CBZ dihydrate was then analyzed by XRPD to investigate the phase transition among CBZ dihydrate, form-III CBZ anhydrate, and form-I CBZ anhydrate, as shown in Figure 3. In general, the dehydration of CBZ dihydrate (8.95 two-theta degrees) resulted in a mixture of form-III CBZ anhydrate (17.02/32.00 two-theta degree) and form-I CBZ anhydrate (9.40/19.92 two-theta degree), and dehydration occurred faster and more thoroughly at higher temperature or for a longer time.

Specifically, at 35 °C, the complete dehydration of CBZ dihydrate was achieved at 60 min, which was confirmed by the absence of 8.95 two-theta peak. The remaining crystals were determined to be a mixture of form-I CBZ anhydrate (9.40 two-theta peak) and form-III CBZ anhydrate (32.00 two-theta peak). For 30 and 45 min, results demonstrate the partial dehydration of CBZ dihydrate into form-I CBZ anhydrate, which was illustrated by the appearance of the peak at 9.40 two-theta degree. Compared to the dehydration kinetics at 35 °C, faster dehydration of CBZ dihydrate was observed at 40 °C, which was illustrated by the disappearance of the CBZ dihydrate two-theta peaks. At 40 °C, CBZ dihydrate was dehydrated into a mixture of form-I CBZ anhydrate (9.40 two-theta peak) and form-III CBZ anhydrate (32.00 two-theta peak) at 30 min. Also, a longer time (45 min) at 40 °C resulted in a crystallinity increase of form-III CBZ anhydrate, which was demonstrated by the increased peak intensities of 17.02/32.00 two-theta peaks. Interestingly, at 35 °C for 60 min or 40 °C for 30 min, most of the dehydrated CBZ dihydrate was obtained as disordered CBZ molecules, which showed the least degree of crystallinity among other samples at higher temperatures. Moreover, at 80 °C, the significant intensity increase of 9.40/19.92 two-theta peaks illustrated that more form-I CBZ anhydrate was obtained following the dehydration of CBZ dihydrate. However, the degree of crystallinity for form-III CBZ anhydrate was slightly reduced, which was confirmed by the decreased intensities of 17.02/32.00 two-theta peaks. In addition, at 44 °C, 50 °C, and 60 °C (Supporting Information Figure S3), CBZ dihydrate was fully dehydrated in as fast as 10 min for 60 °C, and higher temperatures led to a higher degree of crystallinity, which was proven to be form-III CBZ anhydrate (17.02/32.00 two-theta peaks). Meanwhile, the crystallinity of form-I CBZ anhydrate remained unchanged due to the comparable peak intensities of 9.40/19.92 two-theta peaks. Results demonstrate that at a lower temperature (30 °C or 40 °C), the dehydration of CBZ dihydrate resulted in a disordered state of CBZ molecules, which subsequently recrystallized into a mixture of form-III CBZ anhydrate and form-I CBZ anhydrate with a longer time or at a higher temperature. The least degree of crystallinity was achieved at 35 °C for 60 min or 40 °C for 30 min. Additionally, hot stage PLM was conducted to visually study the dehydration process of CBZ dihydrate, as shown in Supporting Information Figure S4. The disappearance of birefringence light from CBZ dihydrate crystals occurred at 80 °C when the sample was heated at a ramp rate of 10 °C/min. The results corresponded to the DSC thermograms of CBZ dihydrate (Figure 1).

### 3.3. CBZ ASDs Prepared by HME using CBZ Dihydrate

#### 3.3.1. Summary of Extrusion Processes

**Figure 4:**
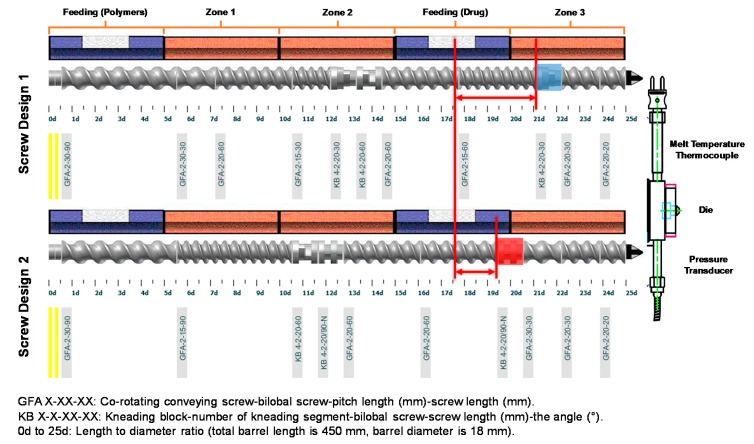
Barrel configurations and screw designs. Screw design 1: 30° and 60° kneading elements in zone 2, and one 30° kneading element (blue) in zone 3; screw design 2: 60° and 90° kneading elements in zone 2, and one 90° kneading element (red) in zone 3.

A Leistritz Micro-18 extruder was configured to enable separate feeding of polymers and drugs, as shown in Figure 4. Five barrels with a total length of 450 mm were used to prepare CBZ ASDs. An endplate and a die of 5 mm in diameter were equipped at the end of the extruder to form the extrudates. Physical blends of Soluplus^®^-Vitamin E succinate (7:3) were fed into the extruder, and subsequently mixed/melted in zone 1 and zone 2. Two kneading elements were used in zone 2 to ensure the homogeneous mixing of Soluplus^®^ and Vitamin E succinate. Also, temperatures of zone 1 and zone 2 were adjusted to fully melt the polymers. Another feeding zone after zone 2 was used to feed drugs. Only zone 3 was applied to mix the molten polymer and the drug for CBZ ASDs preparation. Two screw profiles with different elements were designed. The major difference between these two profiles is shown in Figure 4, indicated by red arrows. Compared to screw deign 1, the distance from the drug feeding zone to the kneading element for mixing (red) was reduced in the screw design 2. Also, a 90° kneading element was used in screw design 2 to provide a better level of mixing between drugs and polymers, while a 60° kneading element was applied in screw design 1. Additionally, a conveying element with a larger pitch was used next to the 90° kneading element (red) in screw design 2, which might reduce the residence time from the drug feeding zone to the die.

As shown in Table 1, CBZ dihydrate and form-III CBZ anhydrate at different drug loadings were processed at various temperatures and screw speeds using two screw designs. Two drug loadings were achieved by changing the drug feed rate and polymer feed rate, while the total feed rate was maintained at 6 g/min. Zone 1 and zone 2 were set at 140 °C for all formulations to ensure that Soluplus^®^-Vitamin E succinate blends were fully melted before feeding the drug. The temperature of the molten polymer measured using an infrared thermometer at the opening of the drug feeding zone was 5–10 °C higher than the set temperature of the drug feeding zone. The temperature measured by the melt temperature thermocouple was consistent with the die temperature for all formulations. The potency of all formulations ranged from 98% to 101%. The torque provided by the extruder was significantly affected by the temperature of the combination of the drug feeding zone, zone 3, endplate, and die. Torque for all formulations varied from 9% at 140 °C (from drug feeding zone to die) to 60% at 60 °C (from drug feeding zone to die). No difference was found in the torque between formulations with CBZ dihydrate or form-III CBZ anhydrate at the same processing condition. The residence time from the drug feeding zone to the die was reduced to 50–60 s for CBZD-F1S/CBZA-F1S compared to around 1.5 min for CBZD-F1/CBZA-F1. In addition, the residence time between formulations using CBZ dihydrate or form-III CBZ anhydrate was comparable.

#### 3.3.2. CBZ ASDs at Different Drug Loading

**Figure 5:**
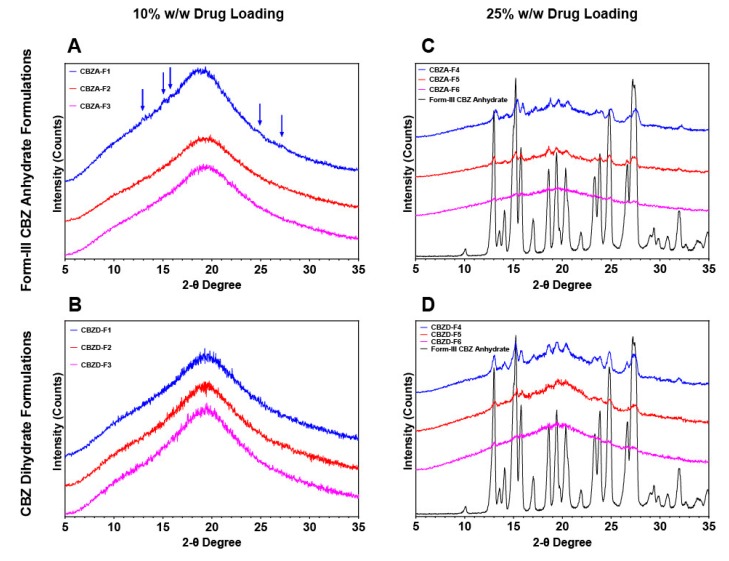
Degree of crystallinity of formulations of CBZ dihydrate and form-III CBZ anhydrate at two drug loadings measured by XRPD. **A**. 10% form-III CBZ anhydrate formulations; **B**. 25% form-III CBZ anhydrate formulations; **C**. 10% CBZ dihydrate formulations; and **D**. 25% CBZ dihydrate formulations.

In this study, two drug loadings, 10% and 25% *w*/*w*, were used to study the effects of CBZ dihydrate on the preparation of CBZ ASDs. All formulations (Table 1) were characterized using various techniques to measure the crystallinity. For 10% drug loading (Figure 5A), crystalline peaks (indicated by the blue arrow) were observed for the CBZA-F1 formulation, which was processed at 60 °C using form-III CBZ anhydrate. At 120 °C and 140 °C, form-III CBZ anhydrate formulations (CBZA-F2 and CBZA-F3) showed the absence of crystalline peaks, which indicated the achievement of CBZ ASDs. However, at 25% drug loading, as shown in Figure 5B, three formulations (CBZA-F4, CBZA-F5, and CBZA-F6) exhibited crystalline peaks, which corresponded to form-III CBZ anhydrate. Additionally, the formulation (CBZA-F6) processed at a higher temperature (140 °C) resulted in less crystallinity. For CBZ dihydrate formulations at 10% drug loading (Figure 5C), three formulations (CBZD-F1, CBZD-F2, and CBZD-F3) processed at 60 °C, 120 °C, and 140 °C showed no crystalline peaks in XRPD experiments. For 25% drug loading with CBZ dihydrate (Figure 5D), due to the presence of the crystalline peak, the formulations (CBZD-F4, CBZD-F5, and CBZD-F6) did not achieve ASDs. However, the intensity of those peaks was relatively less as compared to 25% form-III CBZ anhydrate formulations (Figure 5B), especially for the region between 18 and 21 two-theta degrees.

**Figure 6:**
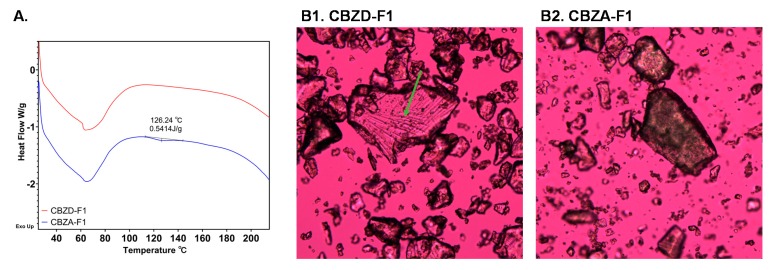
**A**. DSC thermograms of CBZD-F1 (red, 10% CBZ dihydrate) and CBZA-F1 (blue, 10% form-III CBZ anhydrate). **B**. PLM images (100×) of B1. CBZD-F1 and B2. CBZA-F1.

Two formulations, CBZD-F1 and CBZA-F1, were further analyzed using DSC (Figure 6A) and polarized light microscopy (PLM) (Figure 6B1,B2). DSC revealed a small endothermic peak at 126.24 °C for CBZA-F1 formulation. Compared to the DSC of a physical mixture of 10% form-III CBZ anhydrate with 90% processed Soluplus^®^-Vitamin E succinate (Supporting Information Figure S5), this peak corresponded to the undissolved form-III CBZ anhydrate crystals after the extrusion process. CBZD-F1 formulation using 10% CBZ dihydrate exhibited no observable thermal events above 100 °C. Images obtained from PLM provided a higher resolution of crystallinity detection compared to XRPD and DSC. As shown in Figure 6B1, CBZD-F1 was mostly amorphous with some small drug crystals, which showed birefringence light (indicated by the green arrow). For CBZA-F1, a large number of drug crystals was observed (Figure 6B2), and the result corresponded to the observation from DSC and XRPD.

#### 3.3.3. CBZ ASDs Prepared using Different Screw Designs

**Figure 7:**
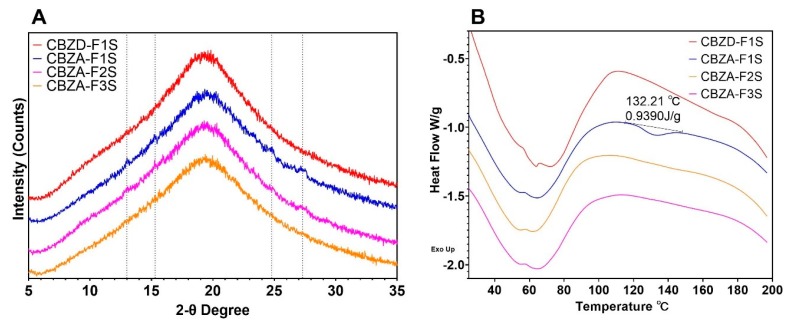
**A**. Degree of crystallinity of formulations using screw design 2 (Grey dot lines indicated the crystalline peaks observed in CBZA-F1S and CBZA-F2S); **B**. DSC thermograms of formulations using screw design 2.

As shown in Figure 4, screw design 2 was used to process CBZ dihydrate and form-III CBZ anhydrate at 10% drug loading (CBZA-F1S, CBZA-F2S, CBZA-F3S, and CBZD-F1S). XRPD results are shown in Figure 7A. CBZA-F1S and CBZA-F2S exhibited crystalline peaks, as indicated by the grey dot lines. Compared to the formulations using screw design 1, no significant difference was found in the degree of crystallinity between formulations except for CBZA-F2/CBZA-F2S. CBZA-F2S (Figure 7A) was processed at 100 °C compared to CBZA-F2 (Figure 5A), which was processed at 120 °C. This processing temperature difference resulted in a higher degree of crystallinity of CBZA-F2S formulation. DSC results (Figure 7B) showed that CBZA-F1S, which was processed at 60 °C, exhibited an endothermic peak at 132.21 °C. This peak was at a higher temperature and had a larger enthalpy compared to CBZA-F1 formulation (Figure 6A). For the other three formulations (CBZD-F1S, CBZA-F2S, and CBZA-F3S), no observable thermal event was found above 100 °C.

**Figure 8:**
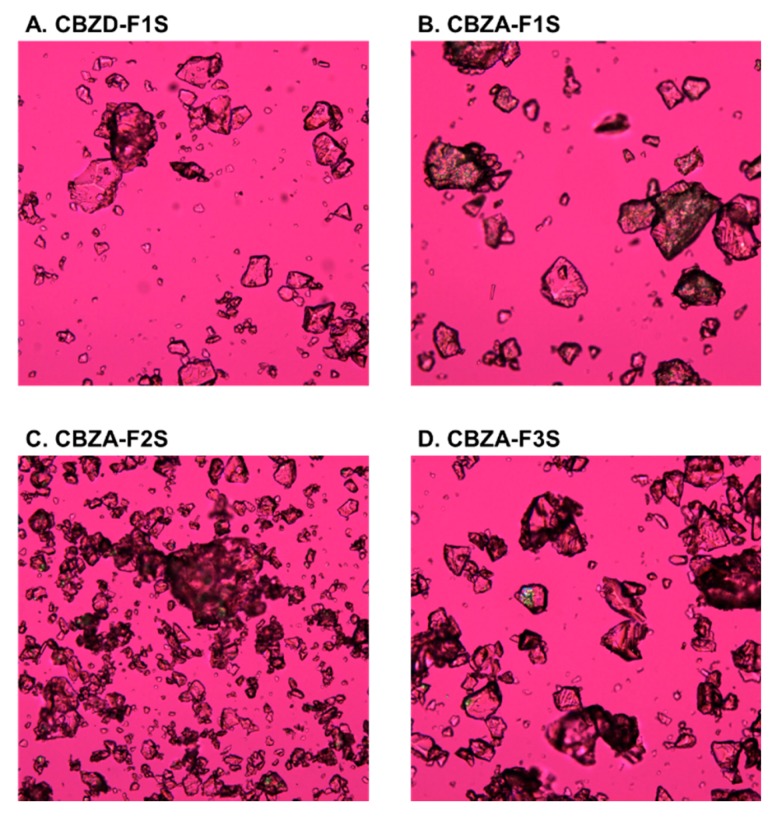
PLM images (100×) of formulations prepared by using screw design 2.

PLM was utilized to further confirm the presence of remaining crystals in those formulations. As shown in Figure 8A, CBZD-F1S of CBZ dihydrate was completely amorphous with the absence of birefringence lights from drug crystals. The other three formulations using form-III CBZ anhydrate (Figure 8B–D) exhibited undissolved drug crystals after the extrusion process. Birefringence lights were also observed in the CBZA-F3S formulation, which was processed at 140 °C.

In summary, CBZ dihydrate formulations (10% drug loading) enabled the preparation of CBZ ASDs at 60 °C using screw design 2, while form-III CBZ anhydrate formulations exhibited undissolved drug crystals at 140 °C. The extrusion processing temperature for CBZ ASDs preparation was significantly reduced by the use of CBZ dihydrate.

### 3.4. In-situ Dehydration Studies of CBZ Dihydrate-polymer System

**Figure 9:**
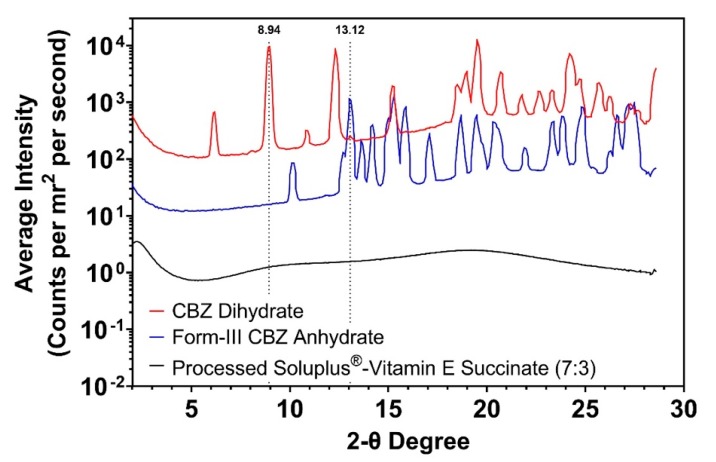
WAXS patterns of CBZ dihydrate (red), form-III CBZ anhydrate (blue), and processed Soluplus^®^-Vitamin E succinate (black). Peak 8.94 two-theta degree and peak 13.12 two-theta degree correspond to CBZ dihydrate and form-III CBZ anhydrate, respectively.

In order to investigate the dehydration of CBZ dihydrate in the polymer matrices (processed Soluplus^®^-Vitamin E succinate), WAXS with temperature controlling unit was utilized. As shown in Figure 9, CBZ dihydrate and form-III CBZ anhydrate exhibited distinctive peaks at 8.94 and 13.12 two-theta degree, respectively. The processed Soluplus^®^-Vitamin E succinate did not exhibit any sharp crystalline peaks through the two-theta range of interest.

**Figure 10:**
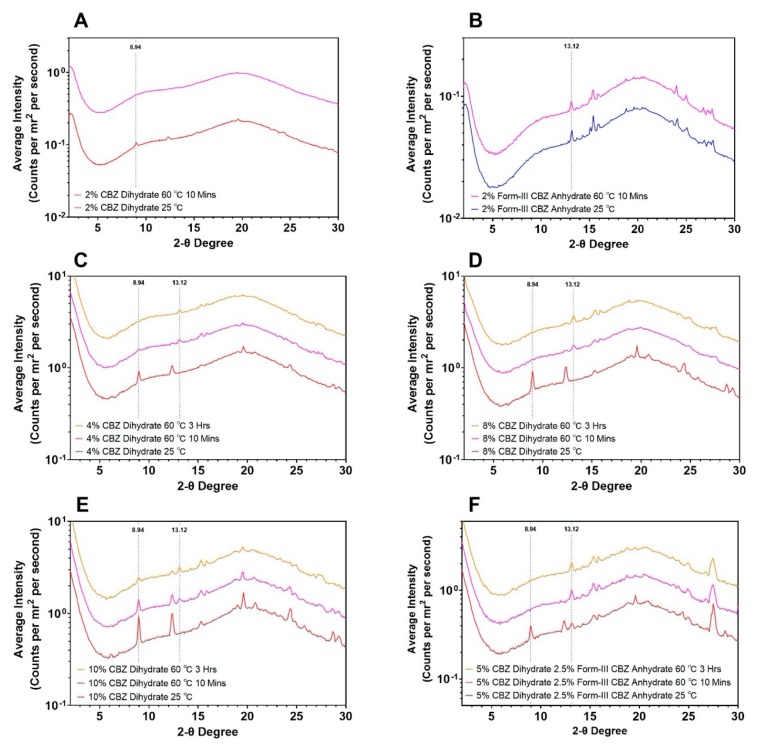
WAXS patterns of CBZ dihydrate and form-III CBZ anhydrate at various experimental conditions. **A**. 2% CBZ dihydrate; **B**. 2% form-III CBZ anhydrate; **C**. 4% CBZ dihydrate; **D**. 8% CBZ dihydrate; **E**. 10% CBZ dihydrate; and **F**. 5% CBZ dihydrate with 2.5% form-III CBZ anhydrate.

The in-situ dehydration studies were conducted at 60 °C from 10 min to 3 h for 5 drug loadings, as shown in Figure 10. To be noted, due to the lack of mixing between the drug and polymer matrices during the WAXS experiments, lower drug loadings (2%, 4%, 5%, and 8%) were selected to study the solid-state transition of CBZ dihydrate upon dehydration in the polymer matrix. Specifically, at 2% drug loading, CBZ dihydrate (Figure 10A) resulted in complete dehydration after being heated at 60 °C for 10 min, which was indicated by the absence of all crystalline peaks including the dihydrate characteristic peak at 8.94 two-theta degrees. The dehydrated CBZ molecules exhibited a WAXS pattern characteristic of amorphous materials, which indicated that the disordered state of CBZ molecules was obtained from the dehydration of CBZ dihydrate [31]. As a comparison, 2% form-III CBZ anhydrate (Figure 10B) showed identical WAXS patterns of the same crystalline peaks before and after heating the sample. The results demonstrate that no change in crystalline structure was observed for form-III CBZ anhydrate. At a higher drug loading of 4% (Figure 10C), after 10 min at 60 °C, the small crystalline peak at 8.94 two-theta degrees indicated that CBZ dihydrate was partially dehydrated, followed by the recrystallization of form-III CBZ anhydrate with a crystalline peak of 13.12 two-theta degree that was observed at 10 min. With continued heating at 60 °C for 3 h, CBZ dihydrate was fully dehydrated with the disappearance of the peak at 8.94 two-theta degrees, and more recrystallized form-III CBZ anhydrate was observed, which was indicated by a higher intensity of peak 13.12 two-theta degree. For 8% and 10% CBZ dihydrate (Figure 10D, E), similar trends were observed as those of the 4% drug loading. Interestingly, at 10% drug loading (Figure 10E), CBZ dihydrate was still not fully dehydrated after 3 h at 60 °C. Additionally, for all CBZ dihydrate samples (Figure 10A,C–E), the peak at 13.12 from the recrystallized form-III CBZ anhydrate exhibited less intensity and sharpness compared to 2% form-III CBZ anhydrate sample (Figure 10B), indicating that some of the CBZ molecules were still in a disordered state or dissolved in the polymer matrices, leading to the partial recrystallization of form-III CBZ anhydrate. Another sample with 5% CBZ dihydrate and 2.5% form-III CBZ anhydrate were heated at 60 °C for 10 min and 3 h (Figure 10F). With the presence of 2.5% form-III CBZ anhydrate, 5% CBZ dihydrate was fully dehydrated and recrystallized into form-III CBZ anhydrate after 10 min. At 3 h, the intensities of all peaks remained identical to the pattern observed at 10 min. These results illustrated that the existence of form-III CBZ anhydrate crystals promoted the dehydration of CBZ dihydrate and the subsequent recrystallization of disordered CBZ molecules resulted from dehydration. In summary, in situ dehydration performed by WAXS demonstrates that the dehydration of CBZ dihydrate led to a disordered state of CBZ molecules, and those molecules can recrystallize into form-III CBZ anhydrate under certain conditions. Moreover, the polymer matrices stabilized or solubilized the disordered CBZ molecules following the dehydration of CBZ dihydrate. In addition, only 2% CBZ dihydrate sample achieved amorphous during the WAXS experiments while CBZ ASDs can be achieved at 10% drug loading in HME. This discrepancy was caused by the lack of mixing between drug and polymers in the WAXS experiments, resulting in drug rich domains, and subsequently, recrystallization of the CBZ molecules.

## 4. Discussion

### 4.1. Extrusion Design

In this study, we conducted dehydration kinetic experiments on neat CBZ dihydrate under various conditions, as discussed earlier in Section 3.2. Based on the dehydration process of CBZ dihydrate, the results illustrate that the disordered state of CBZ molecules following the dehydration was maintained only at a low temperatures (35 or 40 °C). The complete amorphous form cannot be achieved with neat CBZ dihydrate through dehydration. In addition, a physical mixture of 10% CBZ dihydrate with 90% processed Soluplus^®^-Vitamin E succinate (7:3) was analyzed by DSC (Supporting Information Figure S6). Results demonstrate that following the dehydration of CBZ dihydrate, an exothermic crystallization of CBZ molecules was observed at 90 °C followed by the melting of form-III CBZ anhydrate and form-I CBZ anhydrate. Though the processed polymer matrices were added in the physical mixture, the matrices were not fully melted or exhibited high viscosity (Supporting Information Figure S7) below 90 °C. Without aggressive mechanical mixing between the polymer and drug, these viscous or non-molten polymer matrices by themselves were not able to stabilize or solubilize the disordered state of CBZ molecules after the dehydration of CBZ dihydrate, leading to the recrystallization of form-III CBZ anhydrate. Results indicate that during the extrusion process of CBZ dihydrate formulations, the polymer matrices should reach the molten state prior to the dehydration of CBZ dihydrate. Additionally, the extrusion process should provide aggressive and prompt mixing between dehydrated CBZ molecules with the molten polymer matrices in order to achieve CBZ ASDs using CBZ dihydrate. Therefore, we applied a barrel configuration that polymers were fed and melted prior to the feeding of CBZ dihydrate, as shown in Figure 4. Two kneading elements were used in both screw designs in zone 2 to achieve well-mixed polymer matrices. The temperature of zone 3 and zone 5 were maintained at 140 °C throughout the experiments to fully melt the polymer before the drug feeding zone (Table 1). As mentioned earlier, due to this higher temperature of zone 1 and zone 2, the molten polymer exhibited a temperature of 5 to 10 °C higher than the temperature of the drug feeding zone. However, the temperature of the extrudates exiting the extruder was consistent with the die temperature for all formulations. Additionally, a conventional barrel configuration of feeding the polymer and drug together was tested in the preliminary study. No difference was observed between CBZ dihydrate and form-III CBZ anhydrate formulations (results were not shown). The results also demonstrate the hypothesis that the polymer matrices should be in a ready-to-mix state before the dehydration of CBZ dihydrate to achieve CBZ ASDs. Furthermore, two screw designs were studied during the extrusion process. Results confirmed that by using a more aggressive 90° kneading element of screw design 2 and reducing the distance between the drug feeding zone and kneading element, CBZD-F1S was completely amorphous under PLM with the absence of birefringence lights compared to CBZD-F1, while screw design 1 consisted a 60° kneading element and had a longer distance between the drug feeding zone and kneading element. Overall, based on the results from the dehydration kinetics of CBZ dihydrate and extrusion studies of CBZ dihydrate formulations, we hypothesize that the dehydration of CBZ dihydrate will facilitate the mixing between the polymer and disordered CBZ molecules, which enabled the preparation of CBZ ASDs at a much lower temperature (60 °C) compared to the form-III CBZ anhydrate formulations. In order to further explain the effects of CBZ dihydrate on the extrusion process and the possible mechanism for the successful preparation of CBZ ASDs, we performed various experiments discussed in the following section to confirm our hypothesis.

### 4.2. In situ Dehydration of CBZ Dihydrate in HME

As discussed previously, during the heating step in DSC (Figure 1), CBZ dihydrate exhibited a dehydration event at 40 to 75 °C followed by the recrystallization of form-III CBZ anhydrate. Subsequently, the recrystallized form-III CBZ anhydrate showed a phase transition at 145 °C to form-I CBZ anhydrate, which melted at 192 °C. Researchers have demonstrated the successful preparation of amorphous CBZ by dehydrating CBZ dihydrate under certain conditions [28,30,31]. However, those conditions such as low or vacuum pressure, no water vapor pressure, and relatively low temperatures at 40 °C were significantly different from the extrusion process used in our study. In order to fully understand the effects of CBZ dihydrate on the extrusion process and the mechanism of CBZ ASDs preparation, we performed several experiments to study the miscibility between CBZ dihydrate/form-III CBZ anhydrate/form-I CBZ anhydrate with the polymer matrices and the in-situ dehydration of CBZ dihydrate-polymer system using WAXS.

#### 4.2.1. Form-I CBZ anhydrate

Based on the dehydration kinetics of CBZ dihydrate, a mixture of form-I CBZ anhydrate and form-III CBZ anhydrate were obtained under all conditions (Figure 3), which indicated that the successful preparation of CBZ ASDs for CBZ dihydrate formulation might be caused by the dissolution of recrystallized form-I CBZ anhydrate into the polymer matrices. However, form-I CBZ anhydrate exhibited the highest melting point among all CBZ anhydrous forms at 192 °C, which was less susceptible to be solubilized in the polymer matrices at a lower temperature. Additionally, as discussed earlier (Supporting Information Figure S6), with the presence of processed polymer matrices, CBZ dihydrate underwent dehydration followed by the recrystallization of form-III CBZ anhydrate, which was melted at 155.22 °C. Following the melting of form-III CBZ anhydrate, form-I CBZ anhydrate was recrystallized in the polymer matrices and melted again at a higher temperature of 179 °C. Results demonstrate that the recrystallized form-I CBZ anhydrate resulted from dehydration was not able to be solubilized even with the favor of molten polymer matrices until the temperature reached 179 °C, which was significantly higher than the extrusion temperature of 60 °C used for the CBZD-F1S formulation. In addition, Djuris et al. utilized hot stage PLM to study the melt-mediated transformation of form-III CBZ anhydrate into form-I CBZ anhydrate in Soluplus^®^ [47]. Results illustrate that form-III CBZ anhydrate melted at 150 °C and transformed into form-I CBZ anhydrate around 160 °C in the molten Soluplus^®^, which was consistent with our DSC results. Therefore, the recrystallized form-I CBZ anhydrate resulting from the dehydration of CBZ dihydrate was not able to achieve CBZD-F1S formulation, which was processed at 60 °C.

#### 4.2.2. Miscibility of the CBZ Dihydrate-polymer System

Besides the dehydration of CBZ dihydrate, a possible mechanism for the CBZ ASDs preparation was that CBZ dihydrate exhibited better miscibility with the polymer matrices compared to form-III CBZ anhydrate. This advantageous miscibility enabled the solubilization of CBZ dihydrate in the polymer matrices at a lower temperature rather than undergoing dehydration. Several experiments were conducted to study the miscibility of CBZ dihydrate/form-III CBZ anhydrate with the polymer matrices. Two aspects, thermodynamic and kinetic, can contribute to the miscibility between the drug and polymer during the extrusion process [13]. The thermodynamic contribution was analyzed by measuring the IDRs of CBZ dihydrate and form-III CBZ anhydrate in various dissolution media with different concentrations of Soluplus^®^ (Supporting Information Figure S8). According to Wen et al., intrinsic dissolution rate measurement was suitable for accurate determination of critical water activity as well as the free energy of hydration, which were commonly used to characterize the stability of various hydrates thermodynamically [45]. Based on the results from IDR measurements for CBZ dihydrate and form-III CBZ anhydrate, the critical water activity of CBZ dihydrate in 0.5% and 3% Soluplus^®^ dissolution media was identical to the critical water activity of 0.615 in the methanol/water solution. This indicates that the presence of Soluplus^®^ did not change the stability of CBZ dihydrate or the phase boundary between CBZ dihydrate and form-III CBZ anhydrate. Therefore, results demonstrate that neither CBZ dihydrate nor form-III CBZ anhydrate was favored by Soluplus^®^ thermodynamically. Also, compared to the melting peak at 143.90 °C observed in the physical mixture of 10% form-III CBZ anhydrate with the processed polymer matrices (Supporting Information Figure S5), 10% CBZ dihydrate mixtures exhibited a relatively higher melting temperature at 155.22 °C (Supporting Information Figure S6) during in the DSC experiment, which indicated the comparable thermodynamic miscibility of CBZ dihydrate and form-III CBZ anhydrate in the polymer matrices. For the kinetic aspect, rheological properties of CBZ dihydrate and form-III CBZ anhydrate with the polymer matrices were studied. Essentially, the solubilization of drug crystals in polymer matrices is a diffusion-controlled process where the viscosity of the system significantly affects the rate and degree of solubilization [48]. Due to the dehydration of CBZ dihydrate, the dehydrated water molecules may function as an effective plasticizer during the extrusion process, which may significantly lower the viscosity of the polymer system, favoring the solubilization and mixing of drug crystals in the molten polymer. However, the measured complex viscosity of CBZ dihydrate or form-III CBZ anhydrate with polymer matrices was comparable (Supporting Information Figure S7). No significant difference was found under either temperature sweep or angular frequency sweep. Additionally, the dehydrated water molecules from CBZ dihydrate consisted of only 1% of the total weight of the polymer system. Results demonstrate that the dehydrated water molecules were not able to significantly reduce the viscosity of the polymer system to achieve the preparation of CBZ ASDs at 60 °C. In summary, CBZ dihydrate and form-III CBZ anhydrate exhibited comparable miscibility with the polymer matrices.

#### 4.2.3. Disordered State upon the Dehydration of CBZ Dihydrate in Polymer Matrices

In this study, we utilized WAXS with a temperature controlling unit to investigate the in-situ dehydration of a small amount of CBZ dihydrate in the polymer matrices at 60 °C (Figure 10). For 2% CBZ dihydrate mixtures, after 10 min at 60 °C, an amorphous state of CBZ molecules was achieved without the recrystallization of form-III CBZ anhydrate or form-I CBZ anhydrate, which demonstrated our hypothesis that the disordered state of CBZ molecules resulted from the dehydration contributed to the successful preparation of CBZ ASDs at 60 °C for CBZD-F1S formulation. However, with higher CBZ dihydrate content (4%, 8%, and 10%), prompt recrystallization of the disordered CBZ molecules was observed. This was due to the lack of extensive mixing of between amorphous drug molecules and polymer matrices, which resulted in a drug rich domain, subsequently recrystallizing into form-III CBZ anhydrate. Additionally, 2% form-III CBZ anhydrate did not solubilize in the polymer matrices at 60 °C, which further confirmed that the disruption of the CBZ crystalline during dehydration facilitated the extrusion process for preparing CBZ ASDs. Interestingly, as shown in Figure 1, the recrystallization of CBZ molecules following the dehydration occurred at 84 °C, which was 20 °C above the temperature used in WAXS experiments. This lower temperature favored the stability of the amorphous phase that the recrystallization of form-III CBZ anhydrate proceeded slowly and partially, which was indicated by a slow increase of the intensities of form-III CBZ anhydrate crystalline peaks from 10 min to 3 h (Figure 10C–E) and the less sharpness of the crystalline peaks compared to neat form-III CBZ anhydrate (Figure 10B). However, by seeding the system with 2.5% form-III CBZ anhydrate (Figure 10F), the dehydration of CBZ dihydrate and subsequent recrystallization occurred entirely after 10 min at 60 °C. Further heating to 3 h did not result in more recrystallized form-III CBZ anhydrate, which was indicated by the identical crystalline peak intensities. In summary, the dehydration of CBZ dihydrate disrupted the crystalline structure, leading to a disordered state of CBZ molecules. This disordered assembly facilitated the extrusion process to achieve CBZ ASDs at a lower temperature when the polymer matrices were in a ready-to-mix state, and aggressive mixing was provided.

## 5. Conclusions

In this study, we demonstrate a significant decrease in the extrusion temperature from 140 °C to 60 °C when CBZ dihydrate was utilized for CBZ ASD preparation. We report the dehydration of CBZ dihydrate under various conditions and illustrate that the dehydration process of CBZ dihydrate facilitated the extrusion process to achieve CBZ ASDs at a lower temperature. This work provides critical insights about drug form selection, and we demonstrate the importance of proper utilization of drug hydrates to significantly improve the processability of HME for ASD preparation.

## Figures and Tables

**Table 1:** Summary of formulations and extrusion processing parameters used for CBZ ASDs preparation. Drug loading indicates the amount of CBZ on a dry weight basis without hydrated water molecules.

Lot #	Formulation	Total Feed Rate (g/min)	Barrel Temperature (°C)						Screw Design	Screw Speed (rpm)
			Zone 1	Zone 2	Feeding (Drug)	Zone 3	Endplate	Die		
CBZA-F1	10% *w*/*w* form-III CBZ anhydrate 90% *w*/*w* Soluplus^®^-Vitamin E succinate (7:3)	6	140		60				1	100
CBZA-F2					120					
CBZA-F3					140					
CBZA-F4	25% *w*/*w* form-III CBZ anhydrate 75% *w*/*w* Soluplus^®^-Vitamin E succinate (7:3)				60					100
CBZA-F5					120					
CBZA-F6					140					
CBZD-F1	10% *w*/*w* CBZ dihydrate 90% *w*/*w* Soluplus^®^-Vitamin E succinate (7:3)				60					100
CBZD-F2					120					
CBZD-F3					140					
CBZD-F4	25% *w*/*w* CBZ dihydrate 75% *w*/*w* Soluplus^®^-Vitamin E succinate (7:3)				60					100
CBZD-F5					120					
CBZD-F6					140					
CBZA-F1S	10% *w*/*w* form-III CBZ anhydrate 90% *w*/*w* Soluplus^®^-Vitamin E succinate (7:3)				60				2	200
CBZA-F2S					100					
CBZA-F3S					140					
CBZD-F1S	10% *w*/*w* CBZ dihydrate 90% *w*/*w* Soluplus^®^-Vitamin E succinate (7:3)				60					200

## Supplementary Materials

The following are available online at https://www.mdpi.com/1999-4923/12/4/379/s1, Figure S1: Chemical structures of A. carbamazepine, B. Soluplus^®^, and C. Vitamin E succinate, Figure S2: DSC thermograms of CBZ dihydrate prepared using water-suspension (red) or water/ethanol approach (blue), Figure S3: XRPD patterns of dehydrated CBZ dihydrate at various conditions. Three characteristic peaks at 8.95, 17.02/32.00, and 9.40/19.92 two-theta degrees correspond to CBZ dihydrate, form-III CBZ anhydrate, and form-I CBZ anhydrate, respectively, Figure S4: Dehydration of CBZ dihydrate under hot stage PLM, Figure S5: DSC thermograms of the physical mixture of 10% form-III CBZ anhydrate with 90% processed Soluplus^®^-Vitamin E succinate (7:3), Figure S6: mDSC thermograms of physical mixture of 10% CBZ dihydrate with 90% processed Soluplus^®^-Vitamin E succinate (7:3), Figure S7: The complex viscosity of physical mixtures under A. the temperature ramp (at 1 rad/s ang. frequency); and B. the dynamic frequency sweep (at 60 °C testing temperature), Figure S8: IDRs of CBZ dihydrate and form-III CBZ anhydrate in A. methanol/water; B. methanol/water/0.5% Soluplus^®^; C. methanol/water/3% Soluplus^®^. Table S1: Water content and weight loss of CBZ dihydrate and processed Soluplus^®^-Vitamin E succinate measured by KF titration and TGA.
