# Supplementary material for: Influence of Carbamazepine Dihydrate on the Preparation of Amorphous Solid Dispersions by Hot Melt Extrusion
Source: Pharmaceutics. 2020 Apr 20;12(4):379. doi: 10.3390/pharmaceutics12040379 (PMC7238004; doi:10.3390/pharmaceutics12040379)
Supplement: Supplementary file 1 [file pharmaceutics-12-00379-s001.pdf]

pharmaceutics

Supplementary Materials: Influence of Carbamazepine Dihydrate on the Preparation of Amorphous Solid Dispersions by Hot Melt Extrusion

Xiangyu Ma, Felix Müller, Siyuan Huang, Michael Lowinger, Xu Liu, Rebecca Schooler and Robert O. Williams III

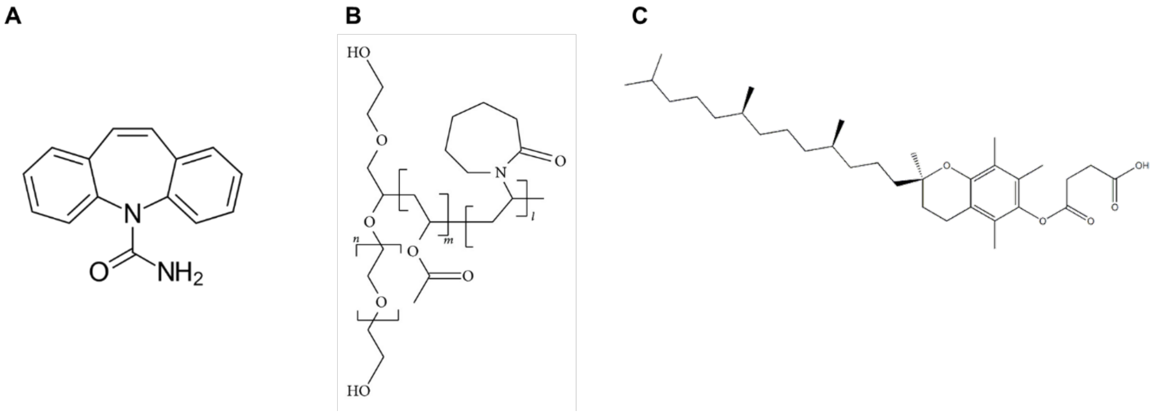

Figure S1. Chemical structures of A. carbamazepine, B. Soluplus®, and C. Vitamin E succinate.

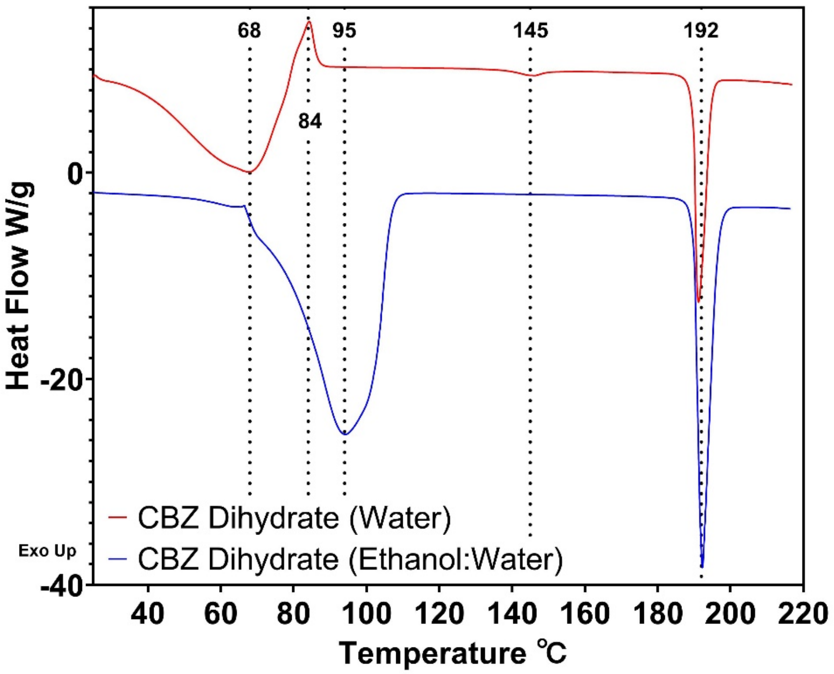

Figure S2. DSC thermograms of CBZ dihydrate prepared using water-suspension (red) or water/ethanol approach (blue).

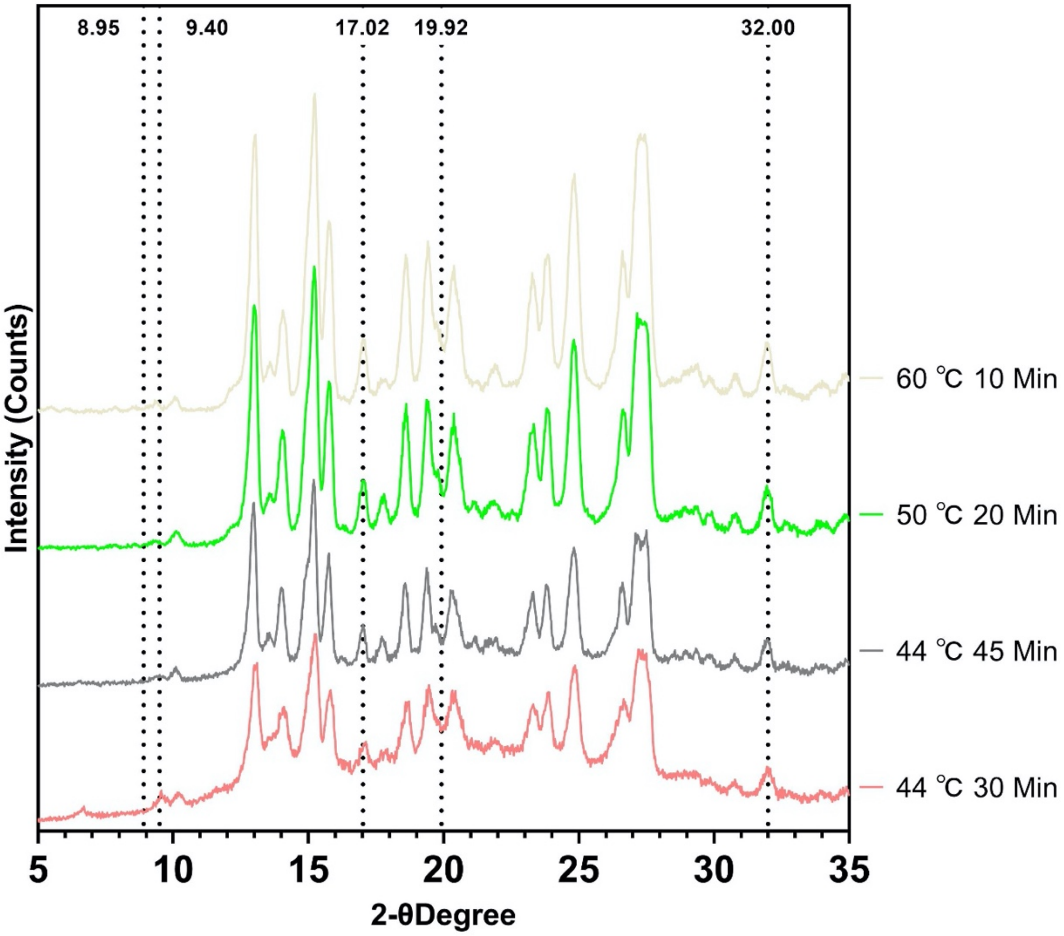

Figure S3. XRPD patterns of dehydrated CBZ dihydrate at various conditions. Three characteristic peaks at 8.95, 17.02/32.00, and 9.40/19.92 two-theta degrees correspond to CBZ dihydrate, form-III CBZ anhydrate, and form-I CBZ anhydrate, respectively.

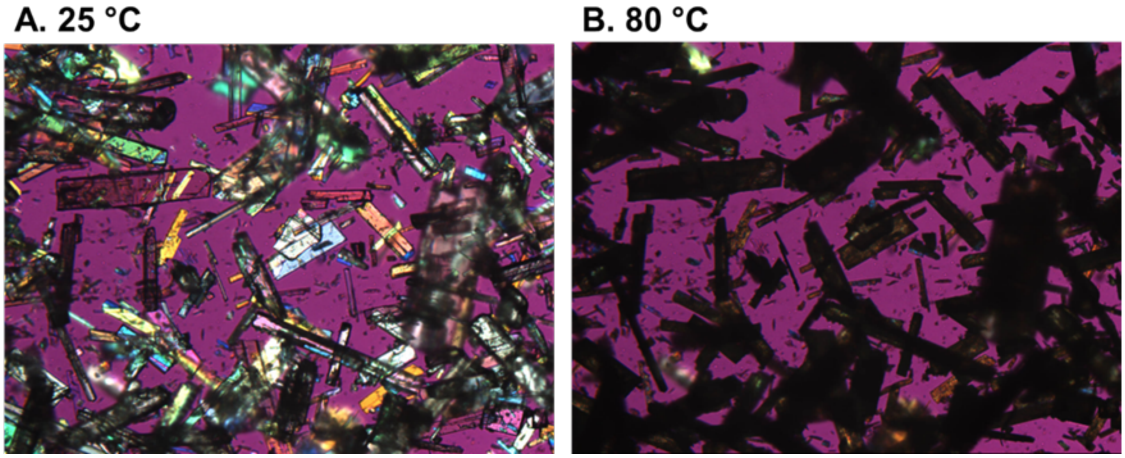

Figure S4. Dehydration of CBZ dihydrate under hot stage PLM.

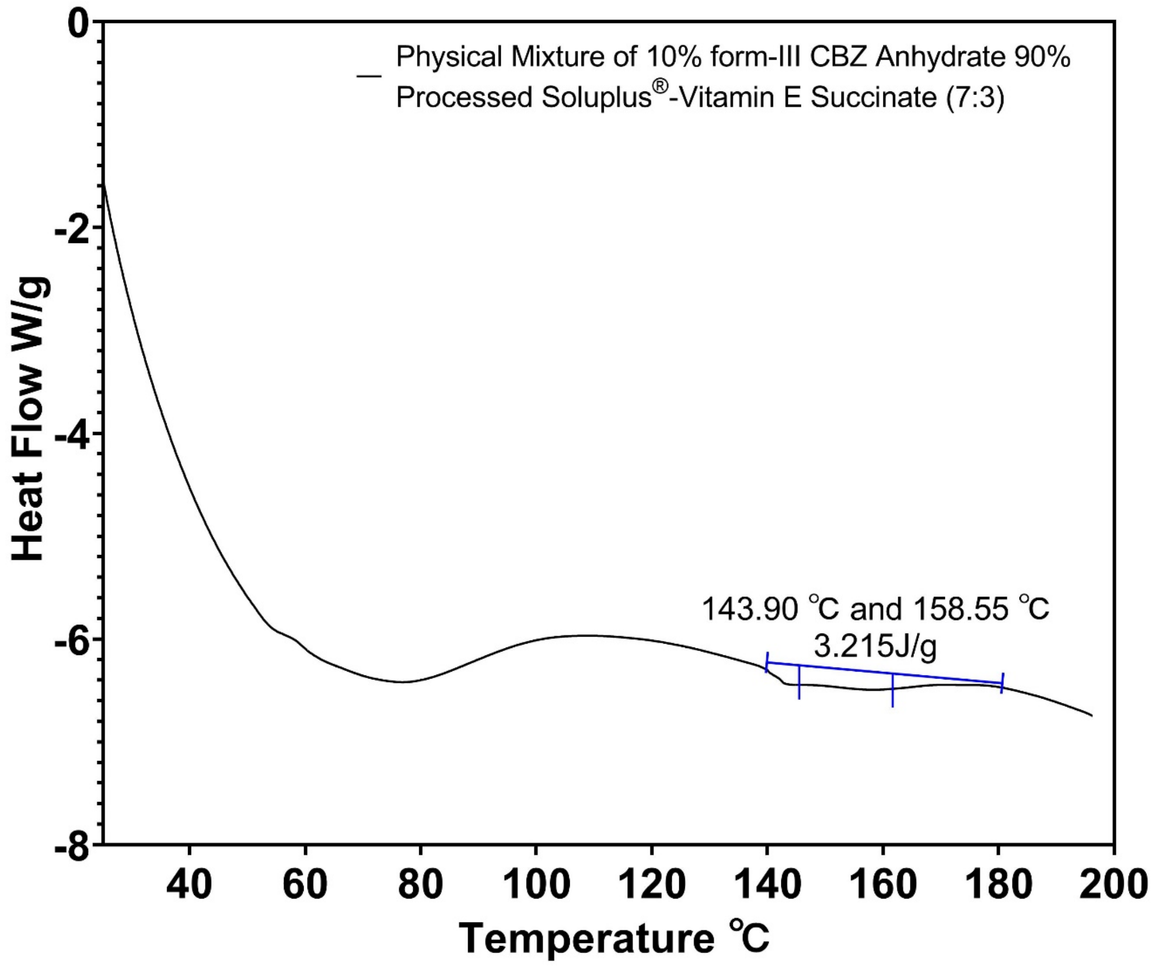

Figure S5. DSC thermograms of the physical mixture of 10% form-III CBZ anhydrate with 90% processed Soluplus®-Vitamin E succinate (7:3).

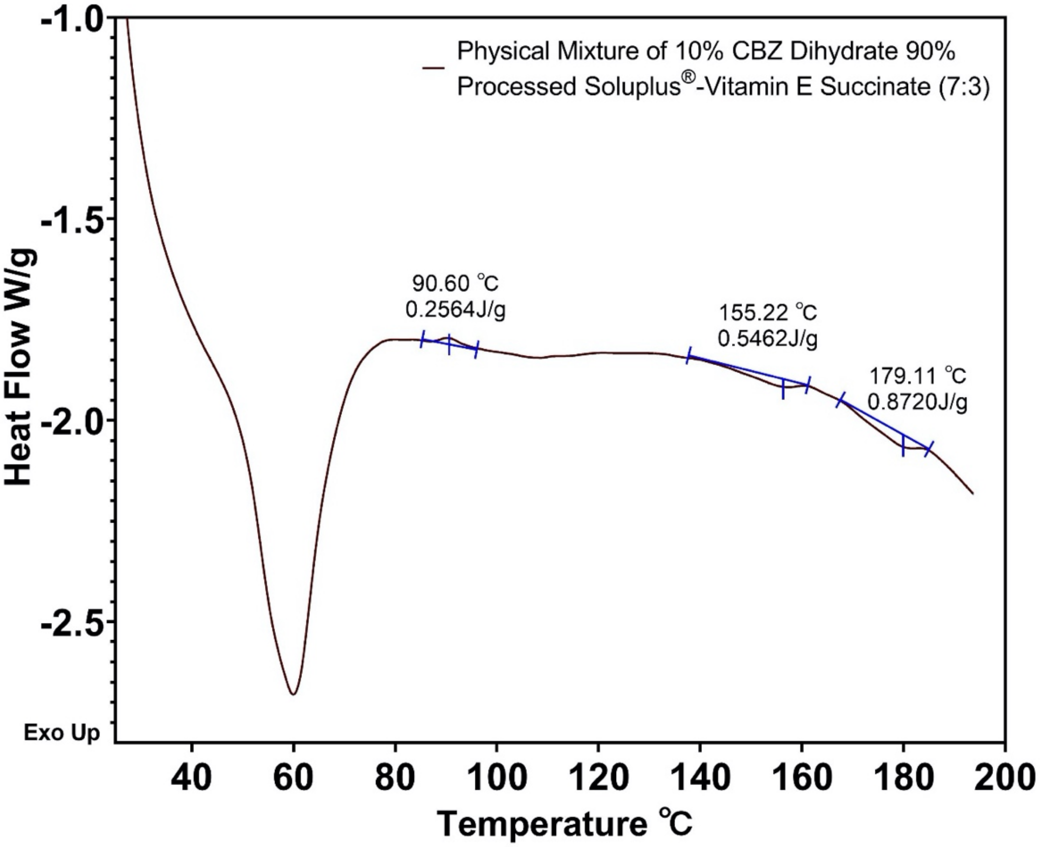

Figure S6. mDSC thermograms of physical mixture of 10% CBZ dihydrate with 90% processed Soluplus®-Vitamin E succinate (7:3).

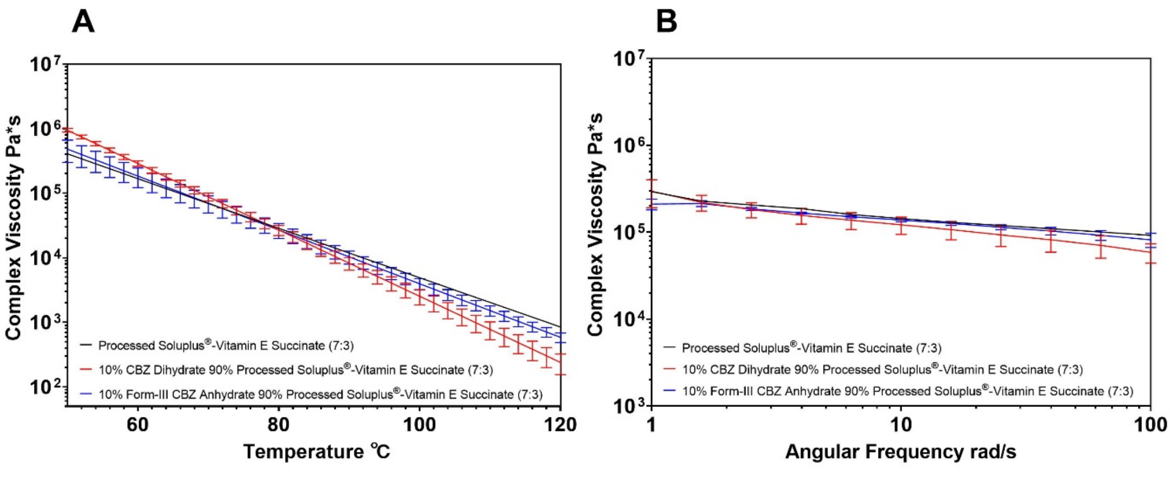

Figure S7. The complex viscosity of physical mixtures under A. the temperature ramp (at 1 rad/s ang. frequency); and B. the dynamic frequency sweep (at 60 °C testing temperature).

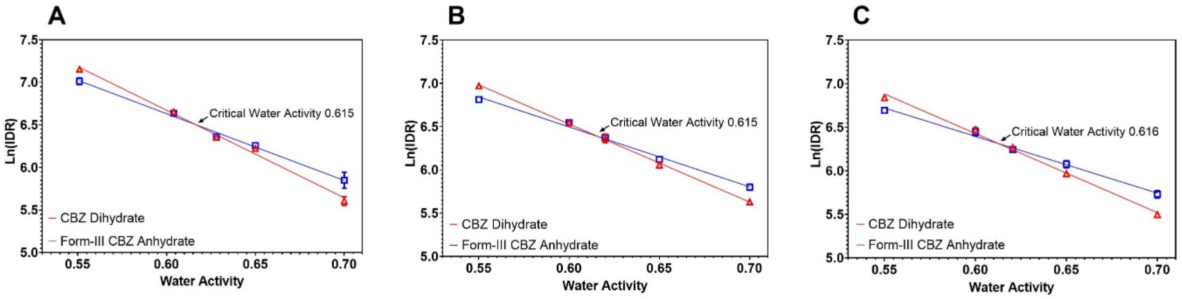

Figure S8. IDRs of CBZ dihydrate and form-III CBZ anhydrate in A. methanol/water; B. methanol/water/0.5% Soluplus®; C. methanol/water/3% Soluplus®.

Table S1. Water content and weight loss of CBZ dihydrate and processed Soluplus®-Vitamin E succinate measured by KF titration and TGA.

Materials	Karl Fischer Titration (Water Content %)	Thermal Gravimetric Analysis (Weight Loss %)
**CBZ Dihydrate**	13.3% ± 0.4%	13.2% ± 0.2%
**Processed Soluplus®-Vitamin E Succinate**	——	Less than 1%
